# Crown roots dominate nitrate uptake in maize (*Zea mays*) under varying soil moisture conditions

**DOI:** 10.1093/aob/mcag197

**Published:** 2026-07-07

**Authors:** Yutong Jiang, Joann K Whalen

**Affiliations:** Department of Natural Resource Sciences, Macdonald Campus of McGill University, Ste-Anne-de-Bellevue, Quebec H9X 3V9, Canada; Department of Natural Resource Sciences, Macdonald Campus of McGill University, Ste-Anne-de-Bellevue, Quebec H9X 3V9, Canada

**Keywords:** Nodal roots, embryonic roots, N stable isotope, root hydraulic conductance, soil N transformation, split-root system, *Zea mays*

## Abstract

**Background and Aims:**

Nitrate (NO_3_^−^) uptake is primarily driven by soil water flow and varies among maize root types. This study quantified NO_3_^−^ uptake by embryonic (primary and seminal) and crown roots under contrasting soil water conditions to determine their relative contributions to total plant N acquisition during early vegetative growth.

**Methods:**

Maize was grown in a split-root pot that segregated the embryonic and crown roots. The sandy-loam soil was moistened to water potentials of either −5 kPa (wet) or −30 kPa (relatively dry). A partial N mass balance was made by destructively sampling shoots, roots, and soils after 0, 24, and 48 h following ^15^N-KNO_3_ injection at the V3 (three-leaf) and V6 (six-leaf) stages. Root hydraulic conductance was quantified using an osmotic-driven root exudation method. Gross nitrification was assessed using a ^15^N isotope dilution technique.

**Key Results:**

At the V3 stage, crown roots had 202 % more N uptake than embryonic roots in wet soil (−5 kPa). However, in relatively dry soil (−30 kPa) N uptake was similar for embryonic and crown roots, and crown root hydraulic conductance was 80 % lower than in wet soil. By the V6 stage, crown roots dominated N uptake, with embryonic roots supplying <20 % of N uptake. Gross nitrification rates did not differ significantly between root types.

**Conclusions:**

Maize NO_3_^−^ uptake depends primarily on the crown roots, due to their capacity to extract water and NO_3_^−^ from soil, even under dry conditions.

## INTRODUCTION

Maize (*Zea mays*) requires nitrogen (N) to produce chlorophyll and protein for maximum growth and optimal yield potential. Maize acquires >90 % of its N supply through mass flow, primarily in the form of nitrate (NO_3_^−^) (Barber, 1962; McMurtrie and Näsholm, 2018). This mass flow mechanism fundamentally couples water and NO_3_^−^ uptake, whereby transpiration-driven water flux through the root system drives NO_3_^−^ movement from soil water into roots and subsequently into xylem vessels connecting to leaf surfaces (Matimati *et al*., 2014; Pou *et al*., 2022).

Continuous water flow creates tension (negative pressure) in the xylem, producing a water potential gradient that draws water and associated solutes, including mobile NO_3_^−^ ions, via both apoplastic (cell wall) and cell-to-cell (symplastic and transcellular) water transport pathways (Steudle and Peterson, 1998). However, NO_3_^−^ delivery via mass flow is constrained in dry soil because reduced water content decreases soil hydraulic conductivity, disrupts hydraulic continuity at root–soil interfaces and reduces root hydraulic conductance, which limits water flux and the associated transport of dissolved NO_3_^−^ from soil to xylem (Ahmed *et al*., 2018*a*). This issue is particularly concerning as agricultural soil water deficit is predicted to intensify under future climate conditions (Plett *et al*., 2020). Therefore, investigating how maize acquires NO_3_^−^ under water-limited conditions requires characterizing their hydraulic properties, which can provide insights for soil water management, ensuring sustainable N uptake in the face of climate change.

Maize forms the primary and seminal roots during embryogenesis and the crown and brace roots during postembryonic development. Each root type exhibits distinct NO_3_^−^ uptake rates and the physical capacity to extract water and dissolved NO_3_^−^ (York *et al*., 2016). Shoot-borne crown roots have ∼63 % more meta- and protoxylem elements and ∼200 % larger xylem vessels than embryonic primary and seminal roots (Tai *et al*., 2016; Hazman and Kabil, 2022). This anatomical structure provides a 5-fold higher axial hydraulic conductance in crown roots, enabling a faster water flow from soil pores to the root xylem (Doussan *et al*., 1998; Ahmed *et al*., 2018*b*). For example, the water uptake velocity was 1.8–2.4 × 10^−5^ cm s^−1^ in crown roots and <5 × 10^−8^ cm s^−1^ in seminal roots of 35-d-old maize (V3–V4 growth stage) (Ahmed *et al*., 2018*b*). This enhanced hydraulic conductance translates to superior NO_3_^−^ uptake capacity in crown roots. Field studies have indirectly shown the crown roots as the dominant tissue for N uptake, showing that maize with fewer but deeper crown roots exhibited enhanced N uptake (Saengwilai *et al*., 2014). In hydroponic environments, maize crown roots showed 22 % higher maximal NO_3_^−^ influx rates (York *et al*., 2016) and more active NO_3_^−^ transporter (Dechorgnat *et al*., 2018) than embryonic roots. However, direct experimental confirmation of crown root dominance in NO_3_^−^ uptake under soil conditions remains lacking, particularly during early vegetative growth. Furthermore, if N uptake varies among the root types, then soil N transformations may vary around embryonic and crown roots due to potential differences in soil pH and the associated microbial community within adjacent soil. To the best of our knowledge, there are no published studies assessing the spatial variability of gross nitrification surrounding the maize root system.

Under dry soil conditions, roots experience reduced delivery of water and solutes (including NO_3_^−^) to their surfaces and respond by adjusting axial and radial hydraulic conductance. As the soil dries, crown roots can show a 50 % reduction in hydraulic conductance, whereas primary and seminal roots show no change after 4 weeks of water deficit (Hazman and Kabil, 2022). Recent research revealed that the radial hydraulic conductivities of the seminal and primary roots decreased immediately when exposed to a mild water deficit and were followed by full recovery in seminal roots and 60 % recovery in primary roots after 4 d of prolonged water deficit in hydroponic conditions (Protto *et al*., 2024). These reductions in axial and radial conductance may be linked to anatomical changes. For example, xylem vessel area in crown roots decreased by up to 80 %, while primary roots showed no change in xylem area (Hazman and Kabil, 2022; Jafarikouhini and Sinclair, 2023). These root-type-specific responses to water deficit may alter how embryonic and crown roots contribute to total plant N uptake during early growth stages, yet this has not been examined in soil-based experiments.

The objective of the present study is to quantify and compare NO_3_^−^ uptake rates by embryonic and crown roots at V3 and V6 growth stages under two contrasting soil water conditions (soil water potential (*Ψ*_soil_) −5 and −30 kPa) using a customized split-root system combined with the ^15^N isotope technique. Our previous study demonstrated that a soil water potential of −30 kPa in sandy loam is sufficient to create a relatively dry condition to reduce root hydraulic conductance compared with wet soil (−5 kPa) while maintaining plant viability (Jiang and Whalen, 2025*a*). This water potential falls within the typical field capacity range for sandy loam soils (−10 to −30 kPa; Lawlor, 2013) and results in limited soil water flow due to the steep hydraulic conductivity curve of this soil type (Cai *et al*., 2022). It is hypothesized here that crown roots have a higher NO_3_^−^ uptake rate than embryonic roots in relatively wet soil (*Ψ*_soil_ = −5 kPa) at both V3 and V6 growth stages due to their higher hydraulic conductance. Under relatively water-deficient conditions (*Ψ*_soil_ = −30 kPa), the relative contribution of embryonic roots to total N uptake is expected to increase due to significant reduction of hydraulic conductance in crown roots. Higher nitrification around crown roots than embryonic roots in the relatively wet soil is also anticipated.

## MATERIALS AND METHODS

### Soil and maize

Soil (0–15 cm) was collected from the Emile A. Lods Agronomy Research Centre in Sainte-Anne-de-Bellevue, Québec (45°25′N, 73°55′W, 39 m elevation) in October 2022 following maize harvest. The soil is a sandy-loam Humic Gleysol of the St Amable series containing 620 g sand kg^−1^, 60 g clay kg^−1^ and 48 g organic C kg^−1^, with a pH of 6.3. Each kilogram of soil (sieved <6 mm) was premixed with NPK fertilizers (expressed as percentage) containing 37 mg urea (46–0–0), 59 mg triple superphosphate (0–46–0) and 28 mg potassium chloride (0–0–60). The maize variety was *Zea mays* cv. PS2790, which was neither genetically modified nor treated with a fungicide or insecticide. All chemicals and reagents were purchased from commercial sources and were at least analytical grade purity.

### Split-root pot design

Experimental split-root pots are designed to segregate the embryonic root system (i.e. primary and seminal roots) from the crown roots (Supplementary Data Fig. S1). An inner chamber (PVC pipe with 5 cm diameter, 25 cm height) was placed in the middle of the outer chamber (PVC pipe with 10 cm diameter, 30 cm height). Field-moist soil was packed into the inner chamber (0.54 kg dry weight basis) and the outer chamber (2.05 kg dry weight basis), with a constant bulk density of 1100 kg m^−3^. An aluminium foil cone (3 cm height) was placed on top of the inner chamber. A small hole (0.5 cm diameter) was pierced on the top of the cone, allowing the primary and seminal roots (i.e. the embryonic roots) to grow through the hole into the inner chamber, while the crown roots were directed to grow in the outer chamber. There was no drainage in the split-root pot to avoid water and soluble nutrient losses by leaching. Visual inspection during destructive sampling confirmed that primary and seminal roots grew exclusively in the inner chamber, while crown roots developed exclusively in the outer chamber.

### Experimental treatments

A two-way factorial experimental design was used to evaluate the N acquisition by maize as influenced by soil moisture (−5 or −30 kPa, measured as soil water potential, *Ψ*_soil_) and maize root type (embryonic roots or crown root), with four replicates per treatment. The maize root types were separated spatially within the split-root pot, and each pot was a replicate in the experiment. The soil water potential was monitored in eight reference split-root pots at two water levels (*n* = 4 for each water level) with a Model MLT tensiometer (Irrometer Inc., Riverside, CA, USA) inserted into the inner chamber and the outer chamber (Supplementary Data Fig. S2). Additional control studies were conducted to confirm that maize root growth in a split-root pot was similar to that in a regular PVC pot (10 cm diameter, 30 cm height) by growing maize in eight pots (2 water levels × 4 replicates) each containing 2.4 kg soil (dry weight basis) and no inner chamber (Supplementary Data Fig. S3).

Maize seeds were pre-germinated in pots (12.7 cm diameter, 15 cm depth) for 10 d before transplanting to the split-root pots. Embryonic roots emerged within 10 d after seeding, but crown roots did not. Thus, embryonic roots were manually directed into the inner chamber through the small hole in the aluminium foil cone, while the crown roots emerged 3–5 d later and grew naturally into the outer chamber. All pots were placed on a growth bench under a fluorescent lighting system (photon flux density 400 µmol^−2^ s^−1^) with controlled light and temperature of 16 h light (25 ± 2 °C) and 8 h dark (18 ± 1 °C). Distilled water was added every 2 d using a syringe with a 12-cm long needle (HaBeuniver Inc., Fuzhou, Fujian, China). The needle was inserted vertically to a soil depth of 10 cm (inner chamber) or 12 cm (outer chamber), ∼2 cm from the tensiometer location to ensure uniform water distribution while avoiding soil disturbance. The syringe was slowly lifted during the water injection process to ensure uniform water distribution while avoiding soil disturbance. Water addition maintained soil water potential at target levels (*Ψ*_soil_ = −5 or −30 kPa) based on tensiometer readings averaged from the eight monitoring pots, and confirmed by the difference in stomatal conductance of maize leaves during the experiment (Supplementary Data Fig. S8). Irrigation continued until 2 d before ^15^N label injection at the V3 and V6 growth stages.

At the V3 and V6 growth stages, N acquisition by each maize root type was evaluated using ^15^N-labelled KNO_3_. When maize had three fully formed leaves (V3), KNO_3_ solution was delivered to achieve a uniform N concentration of 0.02 g N kg^−1^ soil in both water potential treatments. To target at *Ψ*_soil_ = −5 kPa, KNO_3_ solution (0.33 g N L^−1^, 18.4 atom % ^15^N excess) was injected either in the inner (26 mL) or the outer chamber (112 mL) of the pots. To achieve a target of *Ψ*_soil_ = −30 kPa, a different KNO_3_ solution (0.67 g N L^−1^, 18.7 atom % ^15^N excess) was injected either in the inner (13 mL) or the outer chamber (56 mL) of the pots. A non-labelled KNO_3_ solution (0.33 or 0.67 g N L^−1^) was injected into the chamber that was not treated with the ^15^N-KNO_3_ solution, so the total N input was the same for the inner and outer chambers of each pot. Control pots were handled identically except that both inner and outer chambers received non-labelled KNO_3_ solution to establish the natural abundance of ^15^N in maize. Pots (*n* = 56) were destructively sampled at 0, 24 and 48 h post-labelling (Supplementary Data Fig. S4), which allowed sufficient time for diffusion and mass flow to distribute NO_3_^−^ within the soil volume. The pots that were kept until the V6 stage (*n* = 56) received ^14^N-KNO_3_ in the inner and outer chambers at the V3 stage. At the V6 growth stage, these pots were labelled and sampled following the procedure described above for the V3 growth stage.

### Plant sampling and analysis

Following exposure to ^15^N-KNO_3_, pots were destructively sampled by cutting maize stems at the soil surface, then the shoots were rinsed, dried (55 °C for 72 h) and ground (<1 mm). Root excavation was performed carefully to minimize damage. The outer chamber soil was first removed by gentle hand excavation and shaking, exposing crown roots, while removing root-associated soils with a stream of deionized water. The inner chamber was then lifted out intact, and embryonic roots were excavated by gently breaking apart the soil core. A single crown root, seminal root and primary root without obvious damage or breakage from each pot were immediately removed under deionized water for root hydraulic conductance analysis. The rest of the roots were washed thoroughly with deionized water to remove the attached soil particles and then rinsed with 1 mm CaSO_4_ to remove apoplastic ^15^N. Samples were stored in a refrigerator at 4 °C for up to 7 d, while the following analyses were under way.

Root hydraulic conductance was measured within 1 h of sampling by the root exudation method, which measures water flow driven by osmotic gradients in the absence of applied hydrostatic pressure (Knipfer and Fricke, 2011 ). Under these conditions, water movement occurs predominantly via the cell-to-cell pathway (symplastic and transcellular routes) rather than apoplastic bulk flow along cell walls (Steudle and Peterson, 1998). The single primary root, seminal root and crown root were connected to a glass microcapillary tube (0.3-, 0.5-, or 1-mm diameter). Then, the roots sampled from pots with *Ψ*_soil_ = −5 or −30 kPa were bathed in a 0.069 or 4 g L^−1^ sucrose solution. The movement of exudate in the capillary was recorded every 15 min for 3 h and the water flow rate (mm^3^ min^−1^) was calculated as the slope of the linear part of the flow vs time plot. After 3 h, the exudate was collected from the capillary tube using a syringe needle. The osmotic pressures of the bath medium and the xylem exudates were determined by measuring the osmolality using a Model 5520 vapour pressure osmometer (Wescor Vapro, UT, USA). The surface area (m^2^) and length (m) of the single root used for the hydraulic conductance measurement was determined using a Model V700 flatbed photo scanner (Epson Inc., Markham, Canada) and WinRhizo software (Regent Instruments, Quebec, Canada). The root hydraulic conductance (m^3^ s^−1^ MPa^−1^) was calculated using eqn (1):


(1) $$\begin{array}{rl} & \mathrm{Root}\;\mathrm{hydraulic}\;\mathrm{conductance}\\ & \;=((\mathrm{water}\;\mathrm{flow}\;\mathrm{rate}\times 10^{-9}/60)/(\Psi_{\mathrm{xylem}}-\Psi_{\mathrm{medium}}))\\ & \;\;\times (\mathrm{surface}\;\mathrm{area}\;\mathrm{of}\;\mathrm{total}\;\mathrm{roots}/\mathrm{surface}\;\mathrm{area}\;\mathrm{of}\;\mathrm{single}\;\mathrm{roots}) \end{array}$$

where water flow rate is measured in mm^3^ min^−1^ (converted to m^3^ s^−1^ by multiplying by 10^−9^ and dividing by 60), *Ψ*_medium_ is the osmotic pressure of the bath medium (MPa) and *Ψ*_xylem_ is the osmotic pressure of the xylem exudate (MPa). This method measures the actual water transport capacity through the combined effects of both radial and axial pathways in the root system. The surface area scaling factor (surface area of total roots/surface area of single roots) converts the conductance measured on a single excised root to the whole conductance of primary/seminal/crown roots, assuming similar hydraulic properties among the same root types. Root hydraulic conductance values are presented as measurements of the intact root types (m^3^ s^−1^ MPa^−1^) rather than normalized to one unit of surface area (hydraulic conductivity, m s^−1^ MPa^−1^) because our research question focuses on the functional contribution of each root type to whole-plant water and N uptake. Root conductance integrates both the intrinsic hydraulic properties and the morphological characteristics (surface area, length) that determine the actual water extraction capacity of each root type from the soil.

Hand cross-sections of the tip (0–20 mm from root apex), middle (50–70 mm from root apex) and base (0–20 mm from root base) of the crown root, seminal root and primary root were prepared using razor blades for anatomical analyses. Root segments were fixed in 4 % paraformaldehyde in 1× phosphate-buffered saline (PBS) solution while shaking (3 h, 22 °C), and washed three times (30 min, 22 °C) in 1× PBS. The root segments were thin-sectioned, stained with 0.1 % toluidine blue O and washed with 1× PBS solution before sealing with a coverslip. Root anatomy images were taken with a light microscope (Zeiss Imager Z1, Germany) with ×10 magnification. The xylem area, number, diameter, stele area and cross-section area were analysed by ImageJ (Fiji version for Mac OS X, NIH, Bethesda, MD, USA). The cortex area was calculated by subtracting the stele area from the cross-section area (Supplementary Data Fig. S7). The theoretical axial hydraulic conductance of tip (specifically at 15–20 mm from the root apex to ensure presence of mature xylem), middle (50–70 mm from root apex) and base (0–20 mm from root base) segments were calculated (eqn 2) using the Hagen–Poiseuille law (Strock *et al*., 2021) expressed as


(2) $$k=\;\frac{\pi \rho }{128\eta }\overset{n}{\underset{i=1}{\sum }}\;(d_{i}^{4})$$

where *ρ* is the density of xylem sap fluid, assumed to be water (1000 kg m^−3^ at 20 °C), *η* is the viscosity of the xylem sap, assumed to be water (1 × 10^−9^ MPa s at 20 °C) and *d* is the mean diameter of each metaxylem vessel (m). This calculation provides the theoretical axial hydraulic conductance based solely on xylem vessel anatomy.

Morphological traits (length and surface area) of primary, seminal and crown roots were measured by WinRhizo software. Total root length and total surface area also included the excised roots subsampled for root hydraulic and anatomy measurements.

Shoots, embryonic roots and crown roots were oven-dried (at 55 °C for 48 h), weighed (for dry mass) and ground (<1 mm). The N content and ^15^N excess (atom %) of the shoot ([APE]_shoot_), primary root ([APE]_primary_), seminal root ([APE]_seminal_) and crown root ([APE]_crown_) were measured using 2–5 mg of root tissue analysed with an isotope ratio mass spectrometer at the UC Davis Stable Isotope Facility (Davis, CA, USA). The N uptake rate (mg d^−1^) was calculated (eqn 3) according to Barraclough (1995):


(3) $$N\;\mathrm{uptake}=N_{\mathrm{shoot}}\times ([\mathrm{APE}{]}_{\mathrm{shoot}}/[\mathrm{APE}{]}_{\mathrm{KNO}3})+N_{\mathrm{primary}}\times ({\left[\mathrm{APE}\right]}_{\mathrm{primary}}/{\left[\mathrm{APE}\right]}_{\mathrm{KNO}3})+N_{\mathrm{seminal}}\times ([\mathrm{APE}{]}_{\mathrm{seminal}}/[\mathrm{APE}{]}_{\mathrm{KNO}3})+N_{\mathrm{crown}}\times ({\left[\mathrm{APE}\right]}_{\mathrm{crown}}/{\left[\mathrm{APE}\right]}_{\mathrm{KNO}3})$$

where *N*_shoot_, *N*_primary_, *N*_seminal_ and *N*_crown_ are the N contents of shoot, primary roots, seminal roots and crown roots, whereas[APE]_shoot_, [APE]_primary_, [APE]_seminal_ and [APE]_crown_ repesent the ^15^N excess (atom %) of the shoot, primary roots, seminal roots and crown roots, respectively.

### Soil sampling and analysis

Soil from the inner chamber and the outer chamber were removed separately and stored at 4 °C for up to 7 d until analysis. Soil NH_4_^+^ and NO_3_^−^ concentrations were analysed in extracts from 20 g soil mixed with 80 mL 0.5 m K_2_SO_4_ solution (1:4, soil: 0.5 m K_2_SO_4_ solution) and measured calorimetrically at 650 nm on a µQuant microplate reader (BioTek, Winooski, VT, USA) following Sims *et al*. (2008). The ^15^N enrichment of NH_4_^+^ and NO_3_^−^ pools was determined using an acid diffusion procedure (Brooks *et al*., 1989), with >90 % recovery of NH_4_^+^ and NO_3_^−^ from the extracts based on internal standard analysis. Acidified (15 µL of 2.5 m KHSO_4_) glass filter paper discs (5 mm, Whatman GF/D, pre-ashed at 500 °C for 4 h) were sealed in Teflon tape (two discs per Teflon packet). To attain the required 20–50 µg N in solution and considering the low NH_4_^+^ concentration (<2 mg NH_4_^+^ kg^−1^) in soil extracts, 10 mL of soil extract was spiked with 10 mL ^15^N-(NH_4_)_2_SO_4_ (4 mg N L^−1^, 4.2 atom % ^15^N excess) in a 120-mL specimen cup before the diffusion procedure. The ^15^N enrichment of the NO_3_^−^ pool was evaluated after diluting the soil extract with 10 mL of 0.5 m K_2_SO_4_ in a separate 120-mL specimen cup. Diffusion began after adding one Teflon packet and 0.1 g MgO (for ^15^N-NH_4_) or one Teflon packet, 0.1 g MgO, and 0.4 g Devarda’s alloy (for ^15^N-NO_3_) and capping the specimen cup. Blanks (K_2_SO_4_) and the spiking solution (^15^N-(NH_4_)_2_SO_4_) were also analysed to correct for the background ^15^N concentration and ^15^N recovery, respectively. Specimen cups were mixed gently by hand twice a day for 7 d to ensure the reaction of MgO and Devarda’s alloy with the sample solution. After 7 d, filter paper discs were removed from the Teflon packet, dried over concentrated H_2_SO_4_ in a desiccator for ∼48 h and packaged in tin capsules for isotopic enrichment or atom % enrichment (APE) determination on a Micro Cube elemental analyser (Elementar Analysensysteme GmbH, Hanau, Germany) interfaced to a PDZ Europa 20-20 isotope ratio mass spectrometer (Sercon Ltd, Crewe, UK) at the UC Davis Stable Isotope Facility.

### Nitrogen calculations

The atom % ^15^N in the NH_4_-N pool was calculated using eqn (4):


(4) $$\left[\mathrm{APE}{]}_{\mathrm{sample}}\times (N_{\mathrm{extract}}+N_{\mathrm{spike}})=(N_{\mathrm{extract}}\times {\left[\mathrm{APE}\right]}_{\mathrm{NH}4})+(N_{\mathrm{spike}}\times [\mathrm{APE}{]}_{\mathrm{spike}}\right)$$

where [APE]_sample_ is the atom % ^15^N measured from the diffusion discs, *N*_extract_ is the amount of NH_4_-N (µg) in soil extracts, [APE]_spike_ is the atom % ^15^N measured from the diffusion discs from the spike solution, and *N*_spike_ is the amount of NH_4_-N (µg) in the spike (Whalen *et al*., 2001). The unknown in the equation is [APE]_NH4_, the atom % ^15^N in the NH_4_-N pool of soil extracts.

Similarly, atom % ^15^N in the NO_3_-N pool was calculated using eqn (5):


(5) $$\left[\mathrm{APE}{]}_{\mathrm{sample}}\times (N_{\mathrm{NH}4}+N_{\mathrm{NO}3}+N_{K2\mathrm{SO}4})=(N_{\mathrm{NH}4}\times {\left[\mathrm{APE}\right]}_{\mathrm{NH}4})+(N_{K2\mathrm{SO}4}\times {\left[\mathrm{APE}\right]}_{K2\mathrm{SO}4})+(N_{\mathrm{NO}3}\times [\mathrm{APE}{]}_{\mathrm{NO}3}\right)$$

where *N*_NO3_ is the amount of NO_3_-N (µg) in soil extracts, *N*_K2SO4_ is the amount of N in the blank solution and *[APE]*_K2SO4_ is the atom % ^15^N measured from the diffusion discs from the blank solution. The unknown in the equation is [APE]_NO3_, the atom % ^15^N in the NO_3_-N pool of soil extracts.

Gross nitrification rate (mg N kg^−1^ soil d^−1^) was calculated using eqn 6 (Kirkham and Bartholomew, 1954 ):


(6) $$\begin{array}{rl} & \mathrm{Gross}\;\mathrm{nitrification}=([{\mathrm{NO}}_{3}{]}_{t1}\text{–}[{\mathrm{NO}}_{3}{]}_{t2})/(t_{2}\text{–}t_{1})\\ & \;\times (\log([\mathrm{APE}{]}_{{\mathrm{NO}}_{3}-t1}/[\mathrm{APE}{]}_{{\mathrm{NO}}_{3}-t2}))/(\log([{\mathrm{NO}}_{3}{]}_{t1}/[{\mathrm{NO}}_{3}{]}_{t2})) \end{array}$$

where [NO_3_]*_t_*_1_ and [NO_3_]*_t_*_2_ represent the total NO_3_^−^ concentration (mg N kg^−1^) at *t*_1_ (time 1, 0 d) and *t*_2_ (time 2, 7 d), respectively, and [APE]_NO3_*t*1_ and [APE]_NO3_*t*2_ indicate atom % ^15^N excess of NO_3_^−^ at *t*_1_ and *t*_2_, respectively.

### Statistical analyses

For anatomical, morphological and hydraulic measurements, primary and seminal roots are presented separately to reveal type-specific structural differences. For N uptake, they are combined as embryonic roots because both originate from the embryo, share similar developmental timing and occupy the same spatial compartment (inner chamber) in the split-root system, making them indistinguishable when injecting ^15^N tracers. Data were normally distributed (Shapiro–Wilk test, *P* > 0.05) with homogeneous variance (Levene’s test, *P* > 0.05). For each water potential treatment, 20 pots were randomly selected for root morphological measurements (*n* = 20, Fig. 1), 12 pots were selected for root hydraulic measurements (*n* = 12, Fig. 2), regardless of sampling time (0, 24 and 48 h post-labelling), as the analysis of variance revealed no effect (*P* > 0.05) of sampling time on several dependent variables (root length and root hydraulic conductance). The main and interactive effects of root types and soil water potential were evaluated by analysis of variance. When the main effects or interactive effects were significant (*P* < 0.05), mean values were compared with Tukey’s honestly significant test. The xylem number, xylem area and calculated hydraulic conductance under the two water potentials were compared by Student’s *t*-test. Correlation with the Pearson coefficient was used to describe the best-fit lines of the relationship between root hydraulic conductance of each root type and N uptake, using the R 3.6.1 version of R statistical software.

**Fig. 1.**
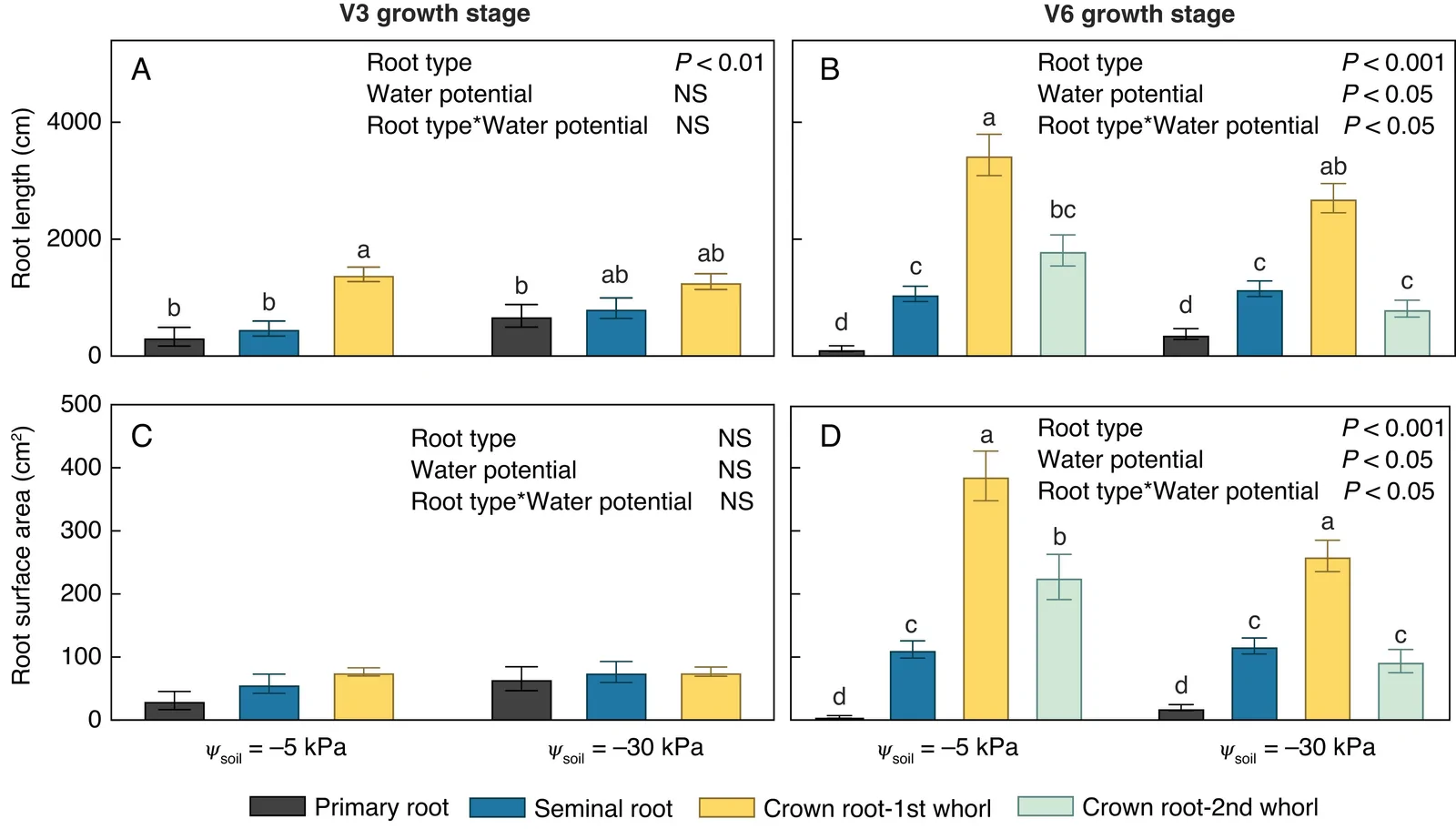
Root morphological traits. (A, B) Root length and (C, D) root surface area of maize grown in the split-root system to the V3 or V6 stage. Maize was grown in pots with a constant water potential (−5 or −30 kPa). Data were pooled among N tracer treatments, and plants with the same water potential were pooled (*n* = 20). Different letters over the bars represent significant differences (*P* < 0.05, Tukey’s honestly significant test). NS, not significant (*P* > 0.05).

**Fig. 2.**
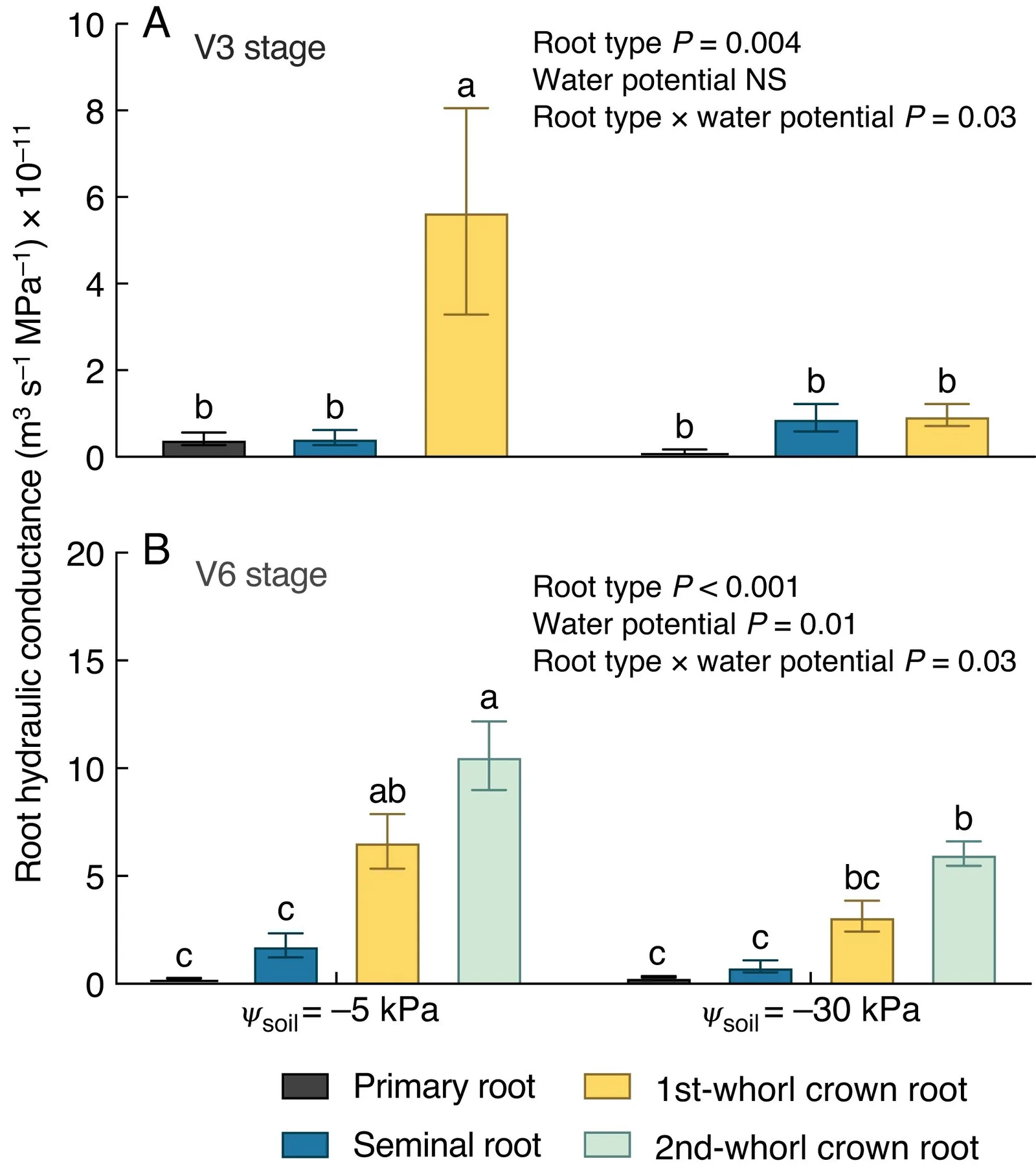
Measured total hydraulic conductance of maize grown in the split-root system. (A, B) Hydraulic conductance at (A) V3 and (B) V6 stage. Maize was grown in pots with a constant water potential (−5 or −30 kPa). Hydraulic conductance was determined with the root exudation method, with one primary, seminal, first-whorl crown root and second-whorl crown root collected from each of the 12 plants grown under one water potential for analysis (*n* = 12). Error bars represent the standard error of the mean. Different letters over the bars represent significant differences (*P* < 0.05, Tukey’s honestly significant test). NS, not significant (*P* > 0.05).

## RESULTS

### Morphology of primary, seminal and crown roots

By the V3 growth stage, crown roots were the largest contributor to total root length, accounting for 63 % of total root length and 46 % of total root surface area in wet soil (*Ψ*_soil_ = −5 kPa; Fig. 1A). In relatively dry soil (*Ψ*_soil_ = −30 kPa), crown roots produced 1276 cm of length and 77 cm^2^ of surface area, which did not differ from primary or seminal roots (*P* > 0.05; Fig. 1A, C). By V6, the crown root system, comprising first- and second-whorl crown roots, constituted 70–80 % of total root length and surface area under both soil water potentials.

### Effect of dry soil on hydraulic conductance of crown roots

At the V3 stage, crown roots had 10 times more hydraulic conductance than primary and seminal roots in wet soil (*P* < 0.05; Fig. 2). However, the hydraulic conductance of crown roots decreased by 83 % in dry soil because root hydraulic conductance interacts with soil water potential (root type × water potential effects, *P* = 0.03). Similar hydraulic conductance in crown, primary and seminal roots in the dry soil were observed (Fig. 2). By the V6 stage, the first- and second-whorl crown roots dominated and contributed 90 % of water-conducting capacity, despite a 37 % reduction in second-whorl crown root hydraulic conductance in dry soil (Fig. 2). The change in hydraulic properties aligned with axial hydraulic conductance, characterized by metaxylem structure. At the V3 growth stage, crown roots had 22 % more xylem vessels and 56–120 % larger xylem area than primary roots, and 75 % more xylem vessels and 84–300 % larger xylem area than seminal roots in wet soil (Table 1). The increased xylem vessel area was associated with the 80–600 % higher axial hydraulic conductance along the crown roots than along the primary and seminal roots. Compared with wet soil, first-whorl crown roots in dry soil developed 35 % less xylem area at the root tip, resulting in 46 % lower axial hydraulic conductance in this region. In contrast, the axial hydraulic conductance was similar in primary and seminal roots growing in the dry soil (*P* > 0.05). At the V6 stage, primary and seminal roots contributed <5 % of axial hydraulic conductance, regardless of soil water condition. The first- and second-whorl crown roots had 16–45 times higher axial hydraulic conductance than the primary and seminal roots. In dry soil, the second-whorl crown roots had 13 % smaller xylem area and 25 % lower axial hydraulic conductance at the root tip region compared with those in wet soil.

**Table 1.** Xylem vessel number, xylem area and axial hydraulic conductance.

Axial root type		Root segment	Xylem vessel number		Xylem area (µm^2^) × 10^4^		Axial hydraulic conductance (kg m MPa^−1^ s^−1^) × 10^−6^	
			−5 kPa	−30 kPa	−5 kPa	−30 kPa	−5 kPa	−30 kPa
V3	Primary	Tip	6 ± 1^b^	5 ± 0^b^	1.13 ± 0.13^b^	1.57 ± 0.15^a^	0.85 ± 0.11^b^	2.08 ± 0.50^a^
		Middle	6 ± 1^b^	6 ± 1^b^	1.66 ± 0.01^b^	1.73 ± 0.18^a^	1.89 ± 0.20^b^	2.31 ± 0.61^a^
		Base	6 ± 1^b^	6 ± 0^b^	2.16 ± 0.40^a^	1.29 ± 0.39^a^	3.26 ± 1.08^a^	1.50 ± 0.95^a^
	Seminal	Tip	5 ± 0^b^	4 ± 0^c^	1.35 ± 0.12^b^	0.86 ± 0^b^	1.56 ± 0.26^b^	0.77 ± 0^b^
		Middle	4 ± 0^c^	4 ± 0^c^	1.28 ± 0.11^b^	1.14 ± 0.18^b^	1.61 ± 0.26^b^	1.34 ± 0.38^b^
		Base	4 ± 0^c^	4 ± 0^c^	0.85 ± 0.23^b^	0.99 ± 0.13^b^	0.85 ± 0.40^b^	1.17 ± 0.17^b^
	Crown (1st whorl)	Tip	8 ± 0^a^	7 ± 1^a^	2.49 ± 0.13^a^	1.62 ± 0.12^a^*	3.4 ± 0.48^a^	1.85 ± 0.35^a^*
		Middle	6 ± 1^b^	6 ± 1^b^	2.92 ± 0.75^a^	2.33 ± 0.28^a^	6.0 ± 2.7^a^	3.44 ± 0.42^a^
		Base	9 ± 1^a^	7 ± 1^a^	3.38 ± 0.39^a^	2.49 ± 0.28^a^	5.9 ± 1.1^a^	3.97 ± 0.72^a^
V6	Primary	Tip	6 ± 0^b^	6 ± 0^b^	1.56 ± 0.12^b^	1.53 ± 0.24^b^	1.6 ± 0.2^b^	1.90 ± 0.58^b^
		Middle	6 ± 0^b^	6 ± 0^b^	1.51 ± 0.19^b^	1.24 ± 0.03^b^	1.6 ± 0.41^b^	1.13 ± 0.05^b^
		Base	6 ± 1^bc^	6 ± 0^b^	1.26 ± 0.26^b^	1.20 ± 0.13^b^	1.02 ± 0.27^b^	1.07 ± 0.20^b^
	Seminal	Tip	4 ± 0^c^	5 ± 0^c^	1.07 ± 0^c^	0.88 ± 0.18^b^	1.43 ± 0^c^	0.71 ± 0.26^b^
		Middle	4 ± 0^c^	4 ± 0^c^	0.55 ± 0.19^b^	0.66 ± 0.07^b^	0.46 ± 0.23^b^	0.44 ± 0.09^b^
		Base	4 ± 0^c^	5 ± 0^c^	0.53 ± 0.22^b^	0.58 ± 0.11^b^	0.45 ± 0.33^b^	0.32 ± 0.10^b^
	Crown (first whorl)	Tip	10 ± 1^a^	8 ± 1^a^	8.77 ± 0.52^a^	4.55 ± 1.92^ab^	23.8 ± 4.6^a^	13.1 ± 6.48^ab^
		Middle	10 ± 2^a^	10 ± 1^a^	7.20 ± 1.90^a^	6.08 ± 1.02^a^	22.4 ± 7.7^a^	16.9 ± 4.6^a^
		Base	10 ± 1^a^	10 ± 2^a^	6.63 ± 1.52^a^	6.07 ± 1.33^a^	18.8 ± 5.9^a^	15.7 ± 4.4^a^
	Crown (second whorl)	Tip	12 ± 1^a^	12 ± 1^a^	8.61 ± 0.28^a^	7.53 ± 0.05^a^*	26.5 ± 2.9^a^	19.9 ± 1.3^a^*
		Middle	12 ± 0^a^	11 ± 0^a^	8.44 ± 0.71^a^	8.12 ± 0.23^a^	25.2 ± 3.1^a^	23.5 ± 1.6^a^
		Base	12 ± 1^a^	11 ± 1^a^	8.93 ± 0.94^a^	8.70 ± 0.44^a^	28.3 ± 5.1^a^	27.4 ± 1.9^a^

Data were determined from three segments (tip, 15–20 mm from root apex; middle, 40–60 mm from root apex; base, 0–20 mm from the base of the stem) of a representative thin-sectioned axial root for each type (primary, seminal and crown, first and second whorls) of maize grown to the V3 or V6 stage in a split-root system. The split-root system was kept at a constant water potential (−5 or −30 kPa).

Values are mean ± standard error, *n* = 4.

Different letters in the same column indicate significant differences (*P* < 0.05, Tukey’s honestly significant test) among the same regions of each root type within the same growth stage. Asterisks (*) indicate significant differences among water potential treatments of each root type within the same growth stage.

### Effects of dry soil on N uptake by crown and embryonic roots

Between 80 and 112 % of the ^15^N was recovered in the soil, shoot and root pools 0, 24 and 48 h after injection of the ^15^N-KNO_3_ solution at V3 (Table 2). Similarly, between 83 and 118 % of the ^15^N was recovered in the soil, shoot and root pools up to 48 h after injection of the labelled solution at V6 (Table 2). Recovery values exceeding 100 % reflect experimental variation around complete recovery from multiple pools, with deviations of <20 % from 100 % representing normal measurement precision limits and sampling variability in ^15^N tracer studies (Whalen *et al*., 2001). Maize shoot and root biomass were progressively enriched with ^15^N in the hours after injection of the ^15^N tracer (Supplementary Data Fig. S5). At the V3 growth stage, N uptake by crown roots was 2.7-fold higher than that by embryonic roots in the wet soil (*P* < 0.05; Fig. 3A). This trend was associated with higher N uptake per unit surface area of crown roots than embryonic roots in the wet soil (Fig. 3C). However, in the dry soil the N uptake rate was similar in crown and embryonic roots (*P* > 0.05), with embryonic roots supplying 38 % of N uptake. By the V6 stage, the crown root system was the dominant pathway for N uptake and was responsible for ∼80 % of N uptake by maize growing in wet and dry soils (Fig. 3B). At this stage, crown roots also maintained a higher N uptake per unit surface area compared with embryonic roots in wet soil, though the difference was less pronounced in the relatively dry soil (Fig. 3D).

**Fig. 3.**
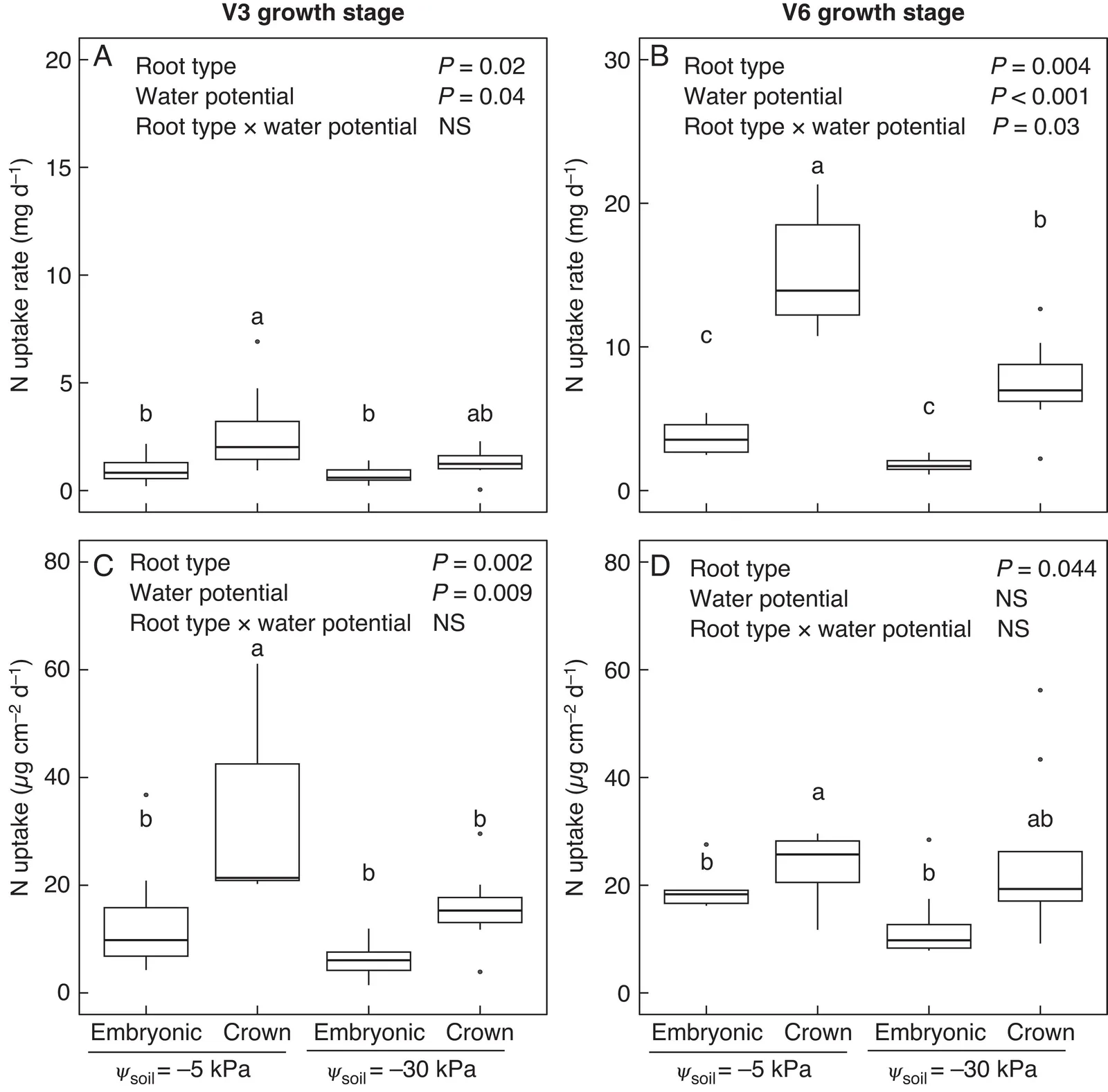
Nitrogen uptake by embryonic roots and crown roots of maize grown in the split-root system. (A, B) N uptake during the V3 (A) and V6 (B) stage. (C, D) N uptake per unit surface area during the V3 (C) and V6 stage (D). Maize was grown in pots with a constant water potential (−5 or −30 kPa). Nitrogen uptake rate was calculated from the ^15^N enrichment in maize shoot after 0, 24 and 48 h ^15^N enrichment periods. At the V3 and V6 stages, one of the chambers, either the inner or outer, received an injection of ^15^N-KNO_3_ solution (18.4 atom % ^15^N excess), while the other chamber received ^14^N-KNO_3_ solution, ensuring exposure of only one root type (either embryonic or crown roots) to ^15^N-KNO_3_ solution. Boxplots show minimum, median and maximum. Dots show outliers. Different letters over bars indicate significant differences (*P* < 0.05, Tukey’s honestly significant test). NS, not significant (*P* > 0.05).

**Table 2.** Percentage of ^15^N from added ^15^NO_3_-N in maize shoot, roots and soil after 0, 24 and 48 h of exposure to ^15^N enrichment*.

Stage	*Ψ*_soil_ (kPa)	Chamber^a^	Time (h)	Shoots (%)	Crown roots (%)	Seminal roots (%)	Primary roots (%)	Soil (%)	^15^N recovery (%)
V3	−5	–	0	0	0	0	0	94 ± 4	94 ± 4
	−30	–	0	0	0	0	0	97 ± 17	94 ± 17
	−5	Inner	24	12 ± 2	2.1 ± 0.4	0.1 ± 0	0.1	66 ± 7	80 ± 6
	−30	Inner	24	13 ± 4	2 ± 0.2	0.5 ± 0.1	0.5 ± 0.1	71 ± 14	88 ± 12
	−5	Outer	24	6 ± 1	0.5 ± 0	0	0.1	88 ± 16	95 ± 15
	−30	Outer	24	4 ± 1	0.4 ± 0	0	0.1 ± 0.1	100 ± 18	105 ± 18
	−5	Inner	48	40 ± 7	5 ± 1	0.4 ± 0.2	0.1 ± 0.1	66 ± 12	112 ± 16
	−30	Inner	48	20 ± 5	2 ± 1	0.9 ± 0.3	0.2 ± 0.1	70 ± 24	93 ± 20
	−5	Outer	48	23 ± 7	0.6 ± 0	0.4 ± 0.2	0	80 ± 26	104 ± 22
	−30	Outer	48	7 ± 1	1 ± 0	0.1 ± 0.1	0.1	103 ± 11	112 ± 10
V6	−5	–	0	0	0	0	0	87 ± 4	87 ± 4
	−30	–	0	0	0	0	0	110 ± 13	110 ± 13
	−5	Inner	24	89 ± 5	9 ± 2	0 ± 0	0.3 ± 0.2	18 ± 4	116 ± 6
	−30	Inner	24	45 ± 7	4 ± 0.4	1 ± 0.5	1 ± 0.5	65 ± 5	118 ± 10
	−5	Outer	24	50 ± 5	25 ± 19	0.1 ± 0	0	15 ± 3	100 ± 22
	−30	Outer	24	30 ± 4	3 ± 0	0.1 ± 0	0	73 ± 16	106 ± 13
	−5	Inner	48	105 ± 3	8 ± 2	0.6 ± 0.1	0.2 ± 0.1	3 ± 1	116 ± 2
	−30	Inner	48	66 ± 4	5 ± 1	0.7 ± 0.1	0.4 ± 0.1	34 ± 11	105 ± 8
	−5	Outer	48	70 ± 4	7 ± 1	0.1 ± 0	0.1 ± 0.1	4 ± 2	83 ± 4
	−30	Outer	48	33 ± 5	3 ± 1	0.3 ± 0.2	0.1 ± 0.1	59 ± 8	95 ± 8

^*^Maize was grown in a split-root system with a constant water potential (−5 or −30 kPa). At the V3 and V6 stages, one of the chambers, either the inner or outer, received an injection of ^15^N-KNO_3_ solution (18.4 atom % ^15^N excess), while the other chamber received ^14^N-KNO_3_ solution, ensuring exposure of only one root type (either the embryonic seminal and primary roots, or the crown roots) to ^15^N-KNO_3_ solution.

Data are mean ± standard error; *n* = 4.

^a^The ^15^N labelling location, either the inner pot or the outer pot of the split-root pot.

The N uptake rate of crown roots was positively correlated with root hydraulic conductance across the soil water potential at both V3 (*r* = 0.752, *P* = 0.001) and V6 stage (*r* = 0.837, *P* < 0.001). In contrast, the N uptake rate of embryonic roots showed no significant relationship with root hydraulic conductance (Fig. 4).

**Fig. 4.**
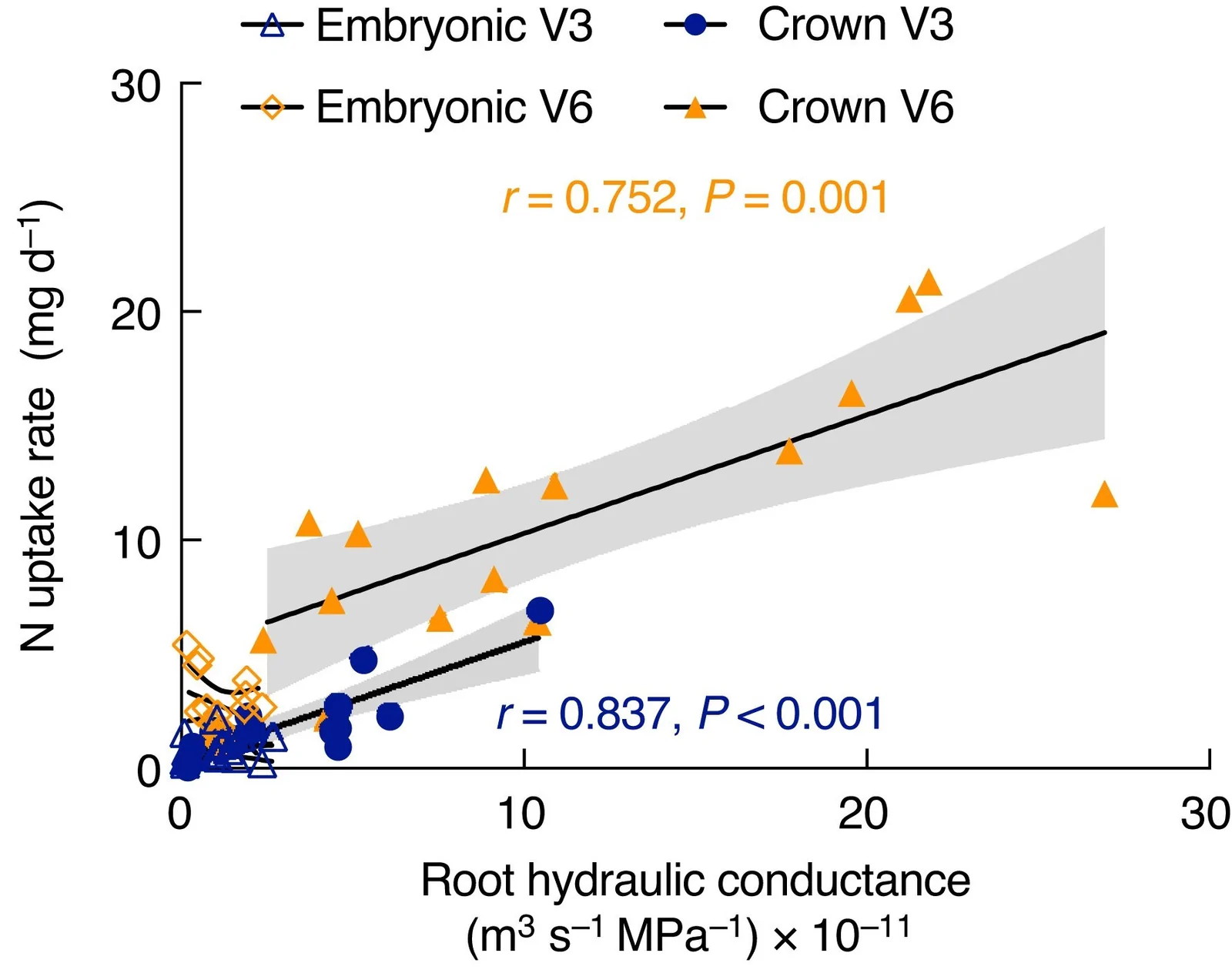
Relationship between maize N uptake rate and root hydraulic conductance of embryonic and crown roots at the V3 and V6 stages. The relationship was described with best-fit lines (with 95 % confidence intervals) when *r*, the Pearson correlation coefficient, was significant (*P* < 0.05). Maize was grown in pots kept at a constant water potential (−5 or −30 kPa). Data collected from the wet and dry soils were pooled; *n* = 16 for each root type.

### Nitrification among the root types

Gross nitrification rates were similar at the V3 and V6 growth stages, ranging from 3.8 to 45.5 mg NO_3_^−^ kg^−1^ d^−1^ (Fig. 5). Root types did not affect the nitrification rate at the V3 and V6 growth stages. The nitrification rate was 330 % higher around crown roots in the wet soil than in the dry soil at the V6 growth stage.

**Fig. 5.**
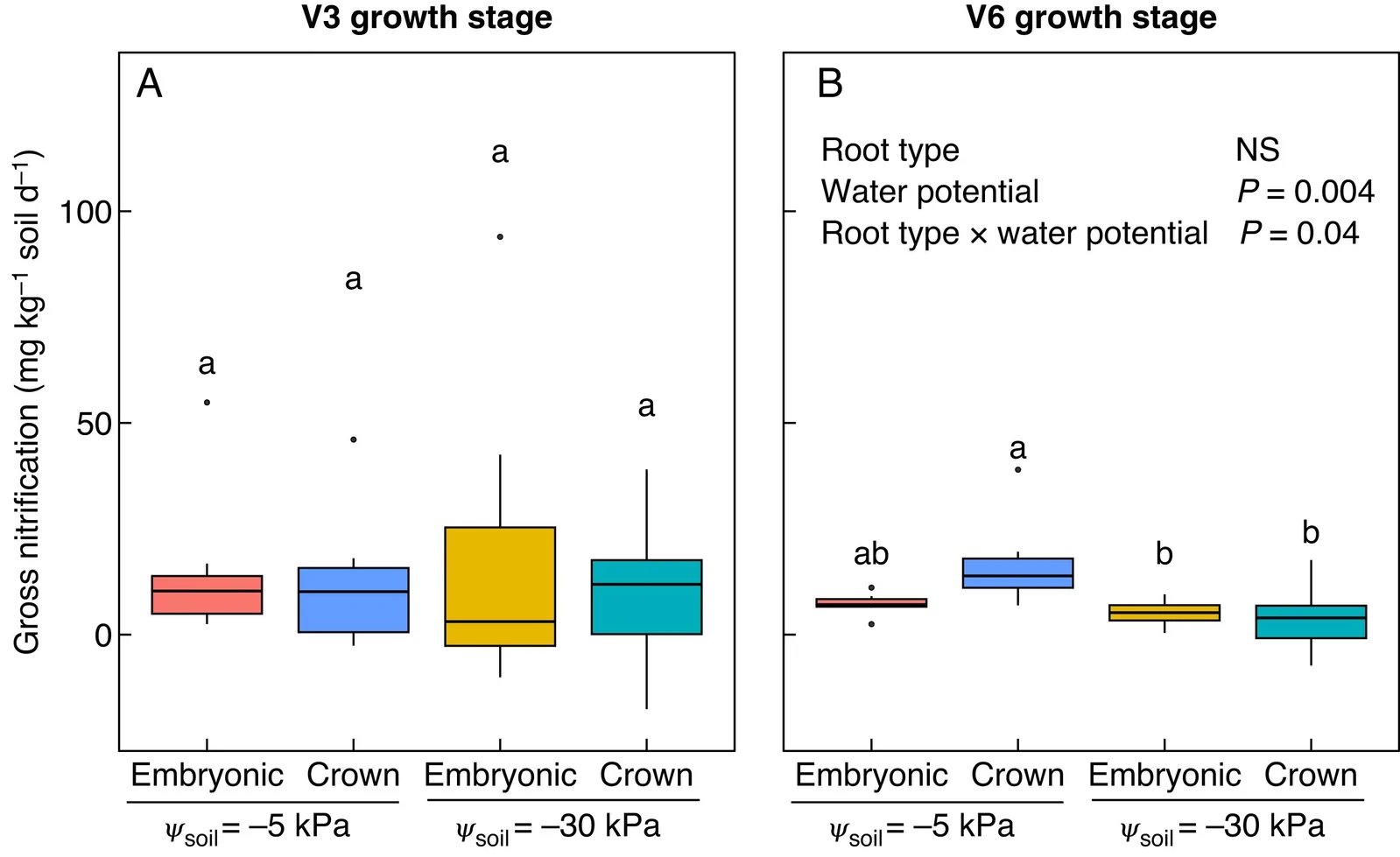
Gross nitrification around embryonic roots and crown roots of maize grown in the split-root system. (A) Nitrification at the V3 stage. (B) Nitrification at the V6 stage. Maize was grown in pots with a constant water potential (−5 or −30 kPa). The gross nitrification rate was calculated from ^15^N isotope dilution after 0, 24 and 48 h ^15^N enrichment periods. At the V3 and V6 stages, one of the chambers, either the inner or outer, received an injection of ^15^N-KNO_3_ solution (18.4 atom %^15^N excess), while the other chamber received ^14^N-KNO_3_ solution, ensuring exposure of only one root type (either embryonic or crown roots) to ^15^N-KNO_3_ solution. Boxplots show minimum, median and maximum. Dots show outliers. Different letters over the bars indicate significant differences (*P* < 0.05, Tukey’s honestly significant test). NS, not significant (*P* > 0.05).

## DISCUSSION

### Crown roots dominate nitrate uptake

Our results support the hypothesis that crown roots have higher NO_3_^−^ uptake than embryonic roots in wet soil at both V3 and V6 (Fig. 3). By V6, crown roots accounted for ∼80 % of total plant N uptake under both −5 and −30 kPa, whereas embryonic roots contributed <20 %. Crown-root dominance reflected both their larger share of root surface area (Fig. 1) and higher N uptake per unit surface area (Fig. 3). Since NO_3_^−^ represented >95 % of the soil mineral N pool (Supplementary Data Fig. S6) and is highly mobile in soil solution, these uptake patterns are consistent with strong coupling between water extraction and NO_3_^−^ delivery to roots under our conditions (Barber, 1962). The greater NO_3_^−^ uptake by crown roots from wet soil (−5 kPa) aligns with a previous report of 1000 times greater water uptake rate in crown roots versus seminal roots of 35-d-old maize (Ahmed *et al*., 2018*b*), suggesting that crown roots can intercept a larger flux of soil solution. Similar functional differentiation between embryonic and post-embryonic root classes has also been reported in cereals such as barley, where nodal roots show 2-fold higher NO_3_^−^ uptake and root-to-shoot translocation capacities than the seminal roots under the hydroponic condition (Liu *et al*., 2020).

The higher N uptake rate by crown roots reflects both their larger contribution to total root surface area (Fig. 1) and their greater N uptake per unit surface area relative to embryonic roots (Fig. 3C, D). Once NO_3_^−^ reaches the root surface, uptake is mediated largely by membrane-localized nitrate transporters, and root-type differences in transporter abundance or activity could contribute to the stronger uptake capacity of crown roots (Jiang and Whalen, 2025*b*). For example, in hydroponic systems crown root tips exhibited higher NO_3_^−^ uptake kinetics compared with seminal root tips, with a 22 % higher maximum NO_3_^−^ influx rate (York *et al*., 2016). Similarly, crown roots showed greater NO_3_^−^ uptake than embryonic roots, attributed to higher expression levels of NO_3_^−^ transporter ZmNPF6.6 in both B73 and F44 inbred lines (Dechorgnat *et al*., 2018). Consistent with these findings, our observation of higher N uptake per unit surface area in crown roots suggests enhanced uptake capacity at the root surface, although this mechanism requires validation in soil-based systems.

### N uptake is consistent with exudation-based hydraulic conductance

Across soil water potentials, N uptake by crown roots was positively correlated with root hydraulic conductance, whereas no such relationship was detected for embryonic roots (Fig. 4). Admittedly, root hydraulic conductance was quantified using the exudation method, which measures osmotic-driven water flow and therefore predominantly reflects the cell-to-cell pathway (symplastic and transcellular routes) under low- or non-transpiring conditions (Steudle and Peterson, 1998). Accordingly, these measurements should be interpreted as comparative indicators of root-type hydraulic capacity, rather than direct estimates under transpiration-driven, hydrostatic conditions.

Under wet soil, crown roots exhibited greater hydraulic conductance than primary and seminal roots (Fig. 2). On average, crown roots of V3 maize had hydraulic conductance of 6 × 10^−11^ m^3^ MPa^−1^ s^−1^ and primary/seminal roots had on average 1.8 × 10^−11^ m^3^ MPa^−1^ s^−1^ (Fig. 2). These values align with previously reported ranges of 0.6 × 10^−11^ to 6 × 10^−11^ m^3^ MPa^−1^ s^−1^ using the root exudation method (Knipfer and Fricke, 2011). Future work using pressure chamber or pressurized flow methods under controlled transpiration would be valuable to quantify apoplastic contributions and test whether root-type rankings persist under hydrostatic driving forces.

Importantly, our main inference, that crown roots dominate N uptake, is supported directly by the split-root ^15^N tracer partitioning, while hydraulic properties provide mechanistic context. The positive correlation between root hydraulic conductance (mainly the cell-to-cell pathway) and N uptake of crown roots suggests a coordinated physiological investment in crown roots, where a higher density or activity of membrane-bound transporters (for both water and NO_3_^−^) facilitates superior resource acquisition compared with embryonic roots (Chaumont and Tyerman, 2014). This may also be due to the anatomical configuration, such as the number of cortical cell layers and the development of apoplastic barriers (e.g. Casparian strips and suberin lamellae) that regulate the radial movement of water (Robbins *et al*., 2014).

While exudation measurements do not quantify radial apoplastic conductance, xylem anatomical traits and Hagen–Poiseuille calculations provide an estimate of theoretical axial conductance capacity. Crown roots exhibited 80–600 % higher theoretical axial conductance than embryonic roots (Table 1), consistent with their larger and more numerous metaxylem vessels. A single crown root produced seven metaxylem vessels, whereas seminal roots produced four metaxylem vessels (Table 1). While these numbers are lower than those reported for the B73 maize inbred line (9–12 vessels in crown roots, 4–6 in primary and seminal roots; Tai *et al*., 2016), the relative difference in xylem diameter between crown and primary/seminal roots (27–68 % larger in crown roots) matches our findings. This greater axial capacity would be expected to facilitate xylem transport under transpiration, although direct measurements under hydrostatic driving forces are needed to quantify apoplastic contributions.

### Relatively dry soil reduces crown-root conductance and N uptake

The second hypothesis, that the contribution of embryonic roots to total N uptake increases in dry soil, was not supported. As soil dried, the embryonic root system contribution to plant N uptake at V3 increased numerically from 25 to 38 %, but this change was not significant (*P* > 0.05; Fig. 3). Exposure to dry soil reduced crown-root hydraulic conductance by 83 % (Fig. 2), whereas primary and seminal roots showed no significant change. This reduction was primarily driven by a 46 % decrease in axial hydraulic conductance at the root tip area (*P* < 0.05; Table 1). Given the positive association between NO_3_^−^ uptake and root hydraulic conductance, reduced conductance in crown roots may limit NO_3_^−^ uptake under dry soil by constraining water flow, NO_3_^−^ delivery and subsequent xylem transport (Henriksson *et al*., 2021). Our result is consistent with Hazman and Kabil (2022), who reported ∼50 % reductions in crown-root hydraulic conductance under water deficit, while embryonic-root conductance was relatively insensitive. By V6, N uptake by crown roots decreased by 52 % in dry soil (Fig. 3B), consistent with previous reports of reduced maize N uptake under reduced soil water availability (Hammad *et al*., 2017; Flynn *et al*., 2023). The significant interaction between water potential and root types on N uptake at the V6 stage (*P* = 0.03) emphasizes the crucial importance of soil water management during this growth stage for optimizing both water and N uptake in maize.

Several mechanisms could explain the reduction in crown-root hydraulic conductance under water deficit. One possibility is that the water deficit leads to decreased root length or surface area, which could affect root hydraulic conductance. In our observations, morphological changes in response to soil water potential were limited to second-whorl crown roots (Fig. 1), suggesting that additional factors are likely involved (Fig. 1). One likely reason is plasticity in xylem anatomy in crown-root tips under water deficit. Metaxylem diameter decreased from 64 to 58 µm in first-whorl crown roots and from 96 to 92 µm in second-whorl crown roots (Table 1). These reductions are smaller than those reported in maize cv. CML538, where root-tip metaxylem diameter decreased from 150 to 30 µm under water deficit (Jafarikouhini and Sinclair, 2023). The diameter of xylem vessels and their plasticity to water deficit vary among maize genotypes, with a wide range of diameters (from 50 to 150 µm) among 44 genotypes (Yang *et al*., 2019), and only two out of four genotypes showed reduced metaxylem number and area under water deficit (Hazman and Kabil, 2022). Together, these findings suggest that genotypes with less plastic xylem responses may better maintain hydraulic function and N uptake under water deficit, warranting further investigation.

### Gross nitrification is similar between root compartments

Our results suggested no significant difference in soil nitrification among root types, contrary to our initial hypothesis (Fig. 5). Nitrification (a biological process controlled by ammonium oxidizers and nitrifiers) is influenced by soil physiochemical properties such as soil moisture and pH. In the present study, the nitrification rates ranged from 18 to 33 mg NO_3_^−^ kg^−1^ d^−1^ in the wet soil and 12–20 mg NO_3_^−^ kg^−1^ d^−1^ in the dry soil (Fig. 4), falling within the reported range of <1–50 mg NO_3_^−^ kg^−1^ d^−1^ (Lteif *et al*., 2010; Zhang *et al*., 2024). The elevated nitrification rate around crown roots in the wet soil compared with the dry soil was consistent with the optimal soil moisture range for nitrification (−5 to −20 kPa, equivalent to 50–80 % of field capacity) as reported earlier (Whalen and Sampedro, 2010). Notably, embryonic roots exhibited higher pH than crown roots (Supplementary Data Fig. S9), likely because crown roots exude more organic acids (Tiziani *et al*., 2022). Nevertheless, soil pH remained within the range of 5.8–7.0, which favours nitrification by autotrophic nitrifiers and results in uniform nitrification among the root types (Whalen and Sampedro, 2010). Further investigation into how soil N transformation varies across diverse root types will contribute to a precise understanding of soil–plant interactions.

### Conclusions

In summary, N uptake in maize depends on the development of crown roots, which have greater hydraulic conductance than embryonic roots. Under dry soil conditions, N uptake and crown root hydraulic conductance decreased at the V3 and V6 stages. The embryonic roots can contribute to maize N uptake in early vegetative growth (up to the V3 stage), but their water and NO_3_^−^-absorbing functions diminish by the V6 stage as the larger, solute-extracting crown root system develops. The present results suggest that soil water management is important to ensure optimal N uptake, especially at the V6 stage and thereafter because the embryonic root functions are negligible.

## Supplementary Material

mcag197_Supplementary_Data

## Data Availability

The data generated and analysed during the current study are provided, in part, as supplementary material and are available from the corresponding author upon reasonable request.

## SUPPLEMENTARY DATA

Supplementary data are available at *Annals of Botany* online and consist of the following. Figure S1: schematic illustration of the split-root system. Figure S2: soil water potential in the inner and outer pots of the split-root system. Figure S3: shoot biomass of maize planted in split-root pots and control pots. Figure S4: experimental setup for ^15^N labelling at V3 and V6 maize growth stages. Figure S5:^15^N enrichment (atom % excess) of maize shoot, crown root, seminal root and primary root after 0, 24 and 48 h of exposure to ^15^N enrichment in the split-root system, at the V3 and V6 stages. Figure S6: soil mineral N (NO_3_^−^ and NH_4_^+^) in the inner and outer chambers of the split-root system after 0, 24 and 48 h exposure to ^15^N enrichment at the V3 and V6 stages. Figure S7: stele area, cortex thickness and stele/cortex ratio of maize grown in the split-root system until V3 and V6 stages. Figure S8: stomatal conductance of maize grown in the split-root system V6 stages. Figure S9: soil pH in the inner and outer chambers of the split-root system after 0, 24 and 48 h exposure to ^15^N enrichment at the V3 and V6 stages.
