# Supplementary material for: Crown roots dominate nitrate uptake in maize (Zea mays) under varying soil moisture conditions
Source: Ann Bot. 2026 Jul 7;138(5):1889–900. doi: 10.1093/aob/mcag197 (PMC13714988; doi:10.1093/aob/mcag197)
Supplement: Supplementary file 1 [file mcag197_supplementary_data.docx]

SUPPLEMENTARY INFORMATION

Crown roots dominate nitrate uptake in maize (*Zea mays* L.) under varying soil moisture conditions

Yutong Jiang^1^, Joann K. Whalen^1^*

^1^Department of Natural Resource Sciences, Macdonald Campus of McGill University

21111 Lakeshore Road, Ste-Anne-de-Bellevue, Quebec, Canada H9X 3V9

*Corresponding author:	Joann K. Whalen

joann.whalen@mcgill.ca,

tel: 001-514-398-7943

**Fig. S1** Schematic illustration of the split-root system. Maize seeds were pre-germinated for about 10 d before transplanting to the split-root pots designed to segregate the embryonic root system (primary and seminal roots) from the crown roots. An inner chamber (PVC pipe with 5 cm diameter, 25 cm height) was placed in the middle of the outer chamber (PVC pipe with 10 cm diameter, 30 cm height). The bottom of split-root pots was covered with aluminum foil to prevent water and soluble nutrient losses.

**Fig. S2** Soil water potential in the inner and outer pots of the split-root system. Tensiometers were inserted into the inner and outer chambers to monitor the soil water potential of the pots. The plants were watered to achieve the target soil water potential (-5kPa or -30kPa) every 2 d, from the first day after transplanting the maize seedling to the split-root system until the V6 stage. No significant difference was detected for soil water potential between the inner and outer pots (*p* > 0.05, Tukey’s honestly significant test). Data represents the mean ± standard error, n = 4.

**Fig. S3** Shoot biomass (A, B) and root length (C, D) were the same (*p* > 0.05, Tukey’s honestly significant test) for maize planted in split-root pots and control pots (PVC pipe with 10 cm diameter, 30 cm height, without an inner chamber). Maize was grown in pots watered to a constant water potential (-5kPa or -30kPa) and was destructively sampled at the V3 and V6 stages. Values are the mean ± standard error, n = 4.

**Fig. S4** Experimental setup for ^15^N labeling at V3 and V6 maize growth stages. Split-root pots received either ^14^N-KNO_3_ or ^15^N-KNO_3_ solutions. To establish initial ^15^N enrichment (0h), one set of pots (n = 8) received ^15^N-KNO_3_ in both chambers and was sampled immediately after injection. For 24h and 48h sampling timepoints, pots received ^15^N-KNO_3_ in one chamber and ^14^N-KNO_3_ in the other chamber. Control pots received ^14^N-KNO_3_ in both chambers to determine natural ^15^N abundance and were sampled at 24h and 48h. Each treatment combination was replicated four times, totaling 56 pots per growth stage.

**Fig. S5** The ^15^N enrichment (atom% excess) of maize shoot, crown root, seminal root, and primary root after 0, 24, and 48 h of exposure to ^15^N enrichment in the split-root system, at the V3 (A) and V6 (B) stages. Maize was grown in pots kept at a constant water potential (-5kPa or -30kPa). At the V3 and V6 stages, one of the chambers, either the inner or outer, received an injection of ^15^N-KNO_3_ solution (18.4% atom ^15^N excess), while the other chamber received ^14^N-KNO_3_ solution, ensuring exposure of only one root type (either embryonic or crown roots) to ^15^N-KNO_3_ solution. Different letters over the bars represent significantly different ^15^N enrichment at the same sampling time (p < 0.05, Tukey’s honestly significant test). Points with error bars represent the mean ± standard error, n = 4. Some data points and lines are missing due to the death of primary and seminal roots by the V6 stage.

**Fig. S6** Soil mineral N (NO_3_^-^ and NH_4_^+^) in the inner chamber and outer chamber of the split-root system after 0, 24, and 48 h exposure to ^15^N enrichment at the V3 and V6 stages. No significant difference was detected for soil mineral N between the inner and outer pots at two water potential (*p* > 0.05, Tukey’s honestly significant test). The inner and outer chambers were kept at a constant water potential (-5kPa or -30kPa). At the V3 and V6 stages, one of the chambers, either the inner or outer, received an injection of ^15^N-KNO_3_ solution (18.4% atom ^15^N excess), while the other chamber received ^14^N-KNO_3_ solution, ensuring exposure of only one root type (either embryonic or crown roots) to ^15^N-KNO_3_ solution. Data was pooled among each chamber type (inner or outer) with the same water potential, regardless of the received KNO_3_ solution type (^14^N-KNO_3_ or ^15^N-KNO_3_). Data points represent the mean ± standard error, n = 12.

**Fig. S7** Stele area, cortex thickness, stele/cortex ratio of maize grown in the split-root system until V3 and V6 stages. Maize was grown in pots watered to a constant water potential (-5kPa or -30kPa). One primary, seminal, 1^st^ whorl crown root, and 2^nd^ whorl crown root were collected from each of the 4 individual plants (n = 4). Error bars represent the standard error of means. Different letters over the bars represent significantly difference (*p* < 0.05, Tukey’s honestly significant test). NS, not significant (*p* > 0.05).

**Fig. S8** Stomatal conductance of maize grown in the split-root system V6 stages. Maize was grown in pots watered to a constant water potential (-5kPa or -30kPa). The stomatal conductance was measured at the mid-blade of the newest fully formed leaf when maize reached V2, V4, and V6 stages. Error bars represent the standard error of means (n = 12). Asterisk (*) indicates a significant difference between the water potential treatments (*p* < 0.05).

**Fig. S9** Soil pH in the inner chamber and outer chamber of the split-root system after 0, 24, and 48 h exposure to ^15^N enrichment at the (A) V3 and (B) V6 stages. The inner and outer chambers were kept at a constant water potential (-5kPa or -30kPa). At the V3 and V6 stages, one of the chambers, either the inner or outer, received an injection of ^15^N-KNO_3_ solution (18.4% atom ^15^N excess), while the other chamber received ^14^N-KNO_3_ solution, ensuring exposure of only one root type (either embryonic or crown roots) to ^15^N-KNO_3_ solution. Data points represent the mean ± standard error, n = 4. Different letters represent significantly different (*p* < 0.05, Tukey’s honestly significant test).
